# When neuroglial tissue wanders: a unique case report of subpleural heterotopia in a triploid foetus and review of the literature

**DOI:** 10.3389/fmed.2025.1598144

**Published:** 2025-09-24

**Authors:** Viktor Procházka, Valeria Skopelidou, Patricie Delongová, Pavel Hurník, Richard Špaček, David Matura, Jan Pavlíček, Andrea Gřegořová, Jana Vaculová, Jozef Škarda

**Affiliations:** ^1^Department of Neurosurgery, Second Medical Faculty, Charles University and Motol University Hospital, Prague, Czechia; ^2^Faculty of Medicine, Institute of Molecular and Clinical Pathology and Medical Genetics, University of Ostrava, Ostrava, Czechia; ^3^Institute of Molecular and Clinical Pathology and Medical Genetics, University Hospital Ostrava, Ostrava, Czechia; ^4^Department of Pathology, EUC Laboratoře CGB a.s., Ostrava, Czechia; ^5^Obstetrics and Gynecology Clinic, University Hospital Ostrava, Ostrava, Czechia; ^6^Department of Pediatrics, Faculty of Medicine, University of Ostrava, Ostrava, Czechia; ^7^Department of Pediatrics, University Hospital Ostrava, Ostrava, Czechia; ^8^Department of Medical Genetics, University Hospital Ostrava, Ostrava, Czechia

**Keywords:** brain heterotopia, neural tube defect, neuroglial heterotopia, Dandy-Walker malformation, triploidy

## Abstract

Heterotopic occurrence of neuroglial tissue is an exceedingly rare phenomenon with unclear pathogenesis, often presenting as lesions outside the central nervous system (CNS). We report a unique case of heterotopic neuroglial tissue identified in the thoracic cavity of a fetus aborted at 14 weeks due to a suspected Dandy–Walker malformation. The fetus exhibited neuroglial tissue growth subpleurally, alongside a suspected Dandy–Walker malformation and ventricular septal defect. While theories such as vascular embolisation, aspiration, and aberrant migration attempt to explain such occurrences, the exact etiology remains elusive. Our case suggests a potential association between triploidy and neuroglial heterotopia, supporting the aberrant migration and differentiation theory.

## Introduction

Heterotopic occurrence of neuroglial tissue is an extremely rare condition, the pathogenesis of which has not been satisfactorily elucidated to date. Outside its usual location, central nervous system (CNS) tissues most commonly manifest as lesions in the head and neck region. In contrast to other neural tube formation disorders, heterotopic neural tissue lacks communication with the subarachnoid space and therefore with the CNS. Symptomatic cases typically present in childhood, while asymptomatic cases may go unnoticed throughout life. There is currently no known association with any neoplasm (1, 2).

In addition to the head and neck, this heterotopic tissue can also be found in the chest or lungs, where it may be the cause of several prenatal and postnatal symptoms. Various clinical presentations are described in the available literature, mostly detailing cases associated with other malformations, neural tube disorders, or anencephalic twins. The pathogenesis of this anomaly is not yet fully understood. Some authors hypothesize (given its relatively frequent occurrence in anencephalic twins) that it is a result of prenatal aspiration of neuroglial tissue. Other possibilities include glial predominant teratomas, hamartomas, and abnormal formation of the neural tube and subsequent migration (3–5).

In the current literature, there have been eight reports of neuroglial heterotopia within the chest cavity, with only one case describing the heterotopia in a subpleural localisation. All of which, including the current case, are summarized in Table 1. For this very reason, we describe another occurrence of this rare nosological entity. In this case report, we present a fetus, aborted due to a Dandy–Walker malformation suspicion at 14 weeks, in whom heterotopic neuroglial tissue was identified in the thoracic cavity. A literature review was included for a better understanding of the subject. The case report itself was processed using the CARE checklist (Supplementary material 1).

**Table 1:** Review of all reported cases describing a chest cavity neuroglial heterotopia in the current literature.

Author	Age	Sex	Symptoms	Localisation of the heterotopic glial tissue	Histopathological findings	Final diagnosis	Treatment	Outcome	Associations
Current case	Fetus	M	-	Right chest cavity, subpleurally	Immature neuroglial tissue, characterized by the direct continuity of immature neuroepithelial structures with the thoracic cavity wall, including focal rosette-like formations	Brain tissue heterotopia	-	Intrauterine asphyxia during induced abortus	Susp. Dandy–Walker syndrome, triploidy (69, XXY), clubfoot deformity, bilateral pulmonary hypoplasia, ventricular septal defect, pulmonary artery hypoplasia
Morgan et al. (3)	Newborn	F	Tachypnoea, retractions, desaturations	Multiple hyperlucent circular lesions in both the lungs	Glial-lined cysts, glial tissue GFAP+, S100+	Pulmonary glial heterotopia	Intensive care for resp. distress	Death on day 22	Anencephalic monoamniotic twin
Dettmer et al. (5)	Newborn	M	Tachydyspnoea, oxygen desaturation	Multiple hyperlucent circular lesions in both lungs	Glial-lined cysts, solid islands of neuroglia GFAP+, S100+	Pulmonary glial heterotopia	Surgical excision (2x)	At 10 weeks readmitted for a nodule in the soft palate – teratoma, excised, otherwise fine	Teratoma with a neural component
Alonso et al. (9)	Newborn	F	Café-au-lait, strabismus, thumb hypoplasia, incurving fifth digit, horseshoe kidney, duodenal membrane, mental retardation, for 3-year-old epilepsy (no respiratory distress intervention), Fanconi anemia	4 cystic nodules- 3 in the right lung, 1 in the left one	Lung tissue replaced by foci of mature glial tissue with psammoma bodies, GFAP+, AE1-3+	Pulmonary glial heterotopia	Bone marrow transplant	Fine (note: author tested DNA – glial tissue was the patient’s, not the twin’s). At 4-year-old alive, well, mild mixed obstructive and restrictive pattern, unchanged nodules	Fanconi anemia, epilepsy
Dixit et al. (20)	1 year	M	Shortness of breath, gradual, progressive	A cyst in the lower lobe of the right lung	Not described	Pulmonary glial heterotopia	Surgical excision	Fine	-
Campo et al. (1)	Newborn	F	APGAR 4,3,2, Hydramnios, cranial malformation, asymmetrical face	Multiple delimited, round, non-prominent nodules on the pleural surface, diffuse, irregularly distributed	Masses of glial cells and nervous fibers, some of which had a central cavity containing aspirated amniotic cells, which were also found elsewhere in the lungs	Pulmonary glial heterotopia	-	Death at 12 min	Cranial malformation, anterior spina bifida
Gonzalez-Crussi et al. (11)	Newborn	F	Respiratory distress, after the resolution, was readmitted at 3 months for respiratory distress again – left lung progression and mediastinal shift to the right	Extensive infiltration of the right lung, grayish-yellow tissue replacing most of the lung parenchyma, similar changes in the left midlung field	Islands of well-differentiated glial tissue replacing all pulmonary structures, neuroglia showed degenerative phenomena.	Pulmonary glial heterotopia	Surgical excision (2x)	Fine with respect to lung function, at 1 year old operated on a cleft palate, excision of fibroadipose mass on the tongue. At 4 years old, moderate mental retardation, delayed receptive and expressive skills	Micrognathia, fusion of tongue to the floor of the mouth, cleft palate (soft and hard), bilateral. Incurving fifth fingers, low-set ears
Rakestraw et al. (18)	Fetus	M	Stillbirth, anencephaly	Two firm gray nodules with a cartilaginous texture in the lungs, subpleurally on both sides	Solid sheets of disorganized neural tissue, separated into nodules, which were neurons and glia	Brain tissue heterotopia	-	-	Anencephaly
Rademaker et al. (19)	Newborn	M	Asphyxia at birth	Light gray, elevated spots on the surface of the lung	Several foci of fine glial fibers, with astrocytes and oligodendrocytes	Heterotopic nervous tissue	-	-	Anencephaly

APGAR, appearance, pulse, grimace, activity, and respiration; DNA, deoxyribonucleic acid; F, female; M, male; min, minutes; mo, months old; w, weeks; yo, years old.

## Case description

**Figure 1:**
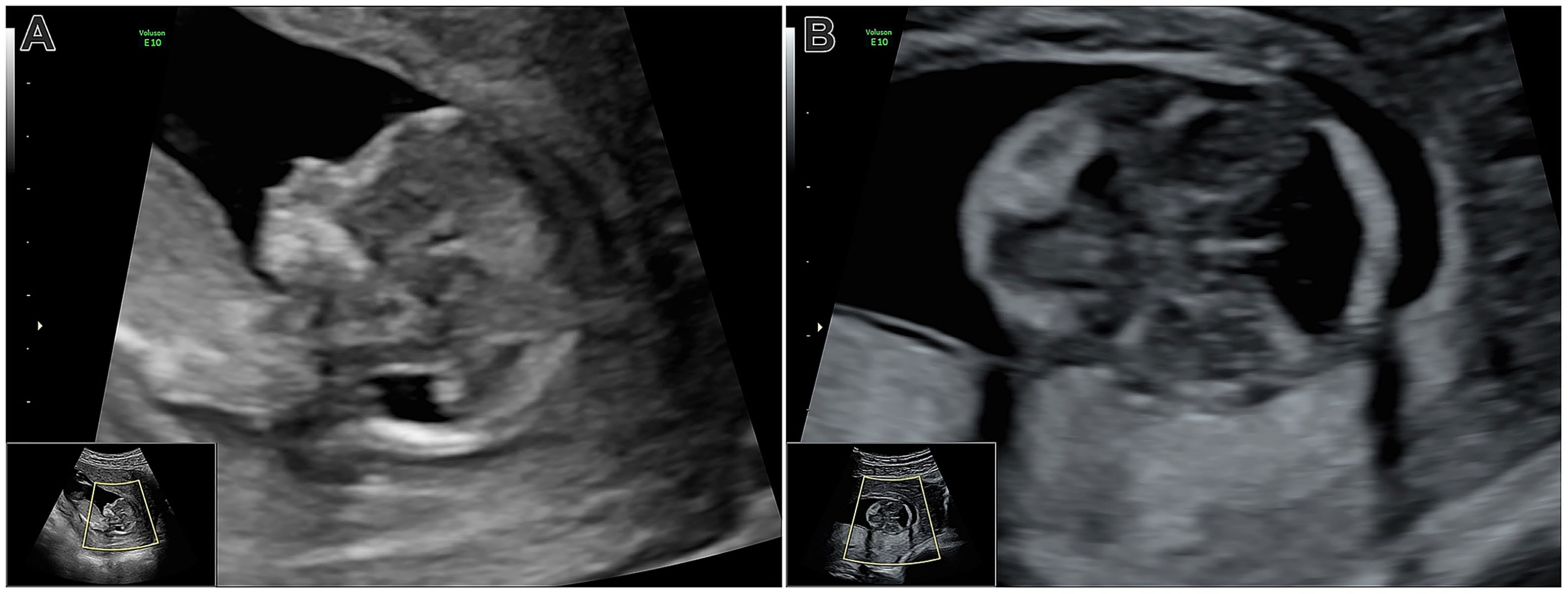
**(A)** Ultrasonography at week 13, mid-sagittal view: Pathological appearance of the posterior cranial fossa, showing the diencephalon and brainstem. The enlarged posterior fossa and fourth ventricle are visible, but intracranial translucency is absent. **(B)** Transverse view of the skull base: The posterior fossa lacks the typical parallel lines of the brainstem and choroid plexus, with fourth ventricle dilation evident.

A 34-year-old woman (gravida 2/para 0) in her second pregnancy was admitted to the Obstetrics and Gynecology Clinic, University Hospital Ostrava, for a scheduled abortion in the 14th gestational week. The first pregnancy resulted in a missed abortion earlier in the same year without any known cause. During the second pregnancy, she had regular antenatal check-ups with no history of epilepsy, diabetes mellitus, hypertension, cardiac disease, bronchial asthma, or blood transfusion. There was no history of consanguinity, family history of malformations, or significant drug intake. The first-trimester screening tests (fb-hCG, PPAP-A, and NT) were also negative. During her abdominal ultrasound, performed as part of a routine check at 13 weeks of gestation, a posterior cranial fossa malformation was detected, resulting in a suspicion of a Dandy–Walker syndrome (Figure 1) and a recommendation for abortion along with a genetic examination. The patient agreed, and an abortion using misoprostol was induced, which finished without the need for a surgical revision.

**Figure 2:**
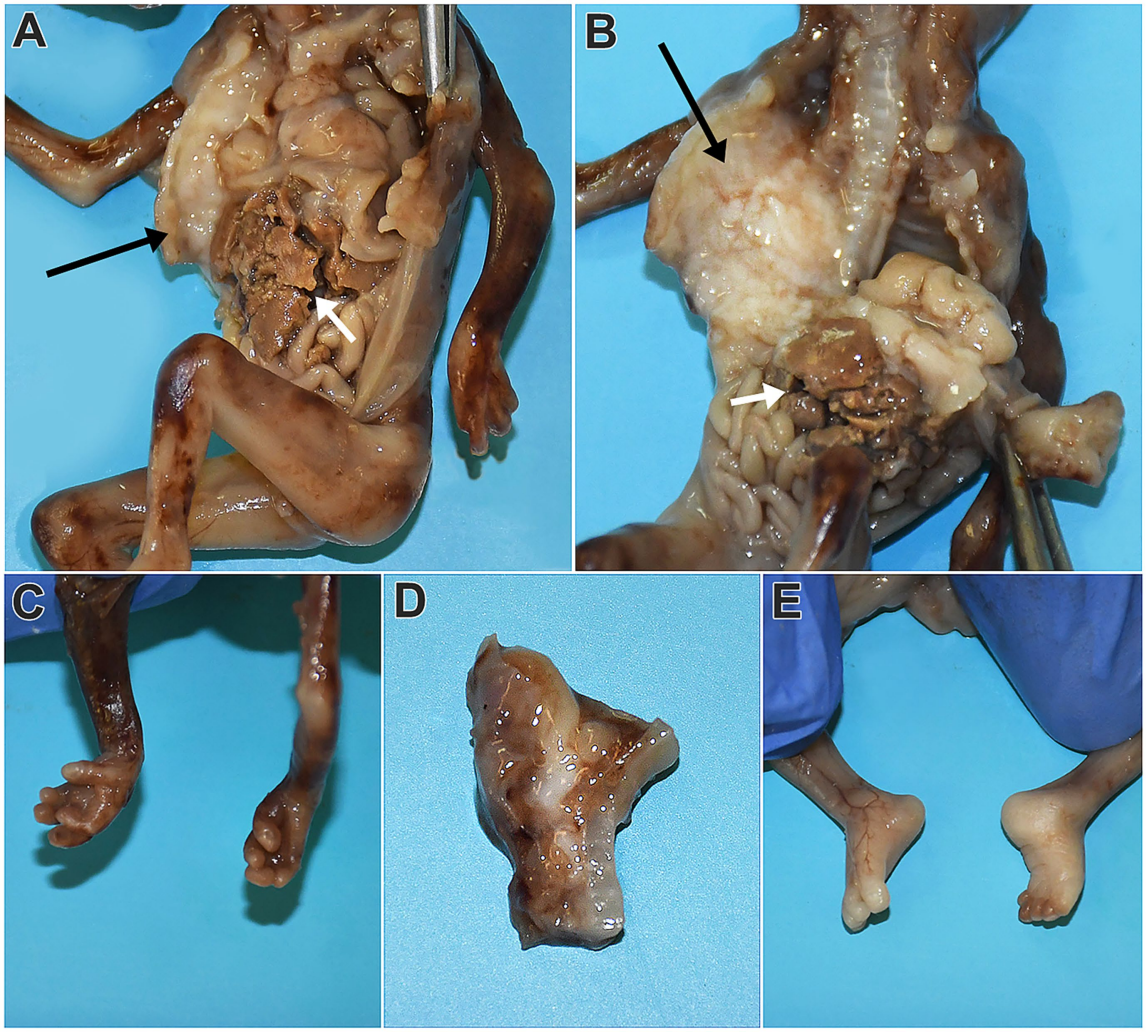
Macroscopic appearance of the fetus following fixation and thoracoabdominal cavity opening. A white mass measuring 1.5 × 1 cm is observed, compressing and displacing the right lung posteriorly and medially (**A**,**B**, neuroglial tissue marked with black arrows, hepatic tissue marked with white arrows). Structures of the dissected skull base with suspected elements of Dandy–Walker malformation are visible in the image **D**. The upper limbs appear consistent with gestational age, with the third digit of the left-hand crossing over the second digit. However, the fingers are not fused **(C)**. The lower limbs display signs of clubfoot deformity, with no other pathological findings noted **(E)**.

**Figure 3:**
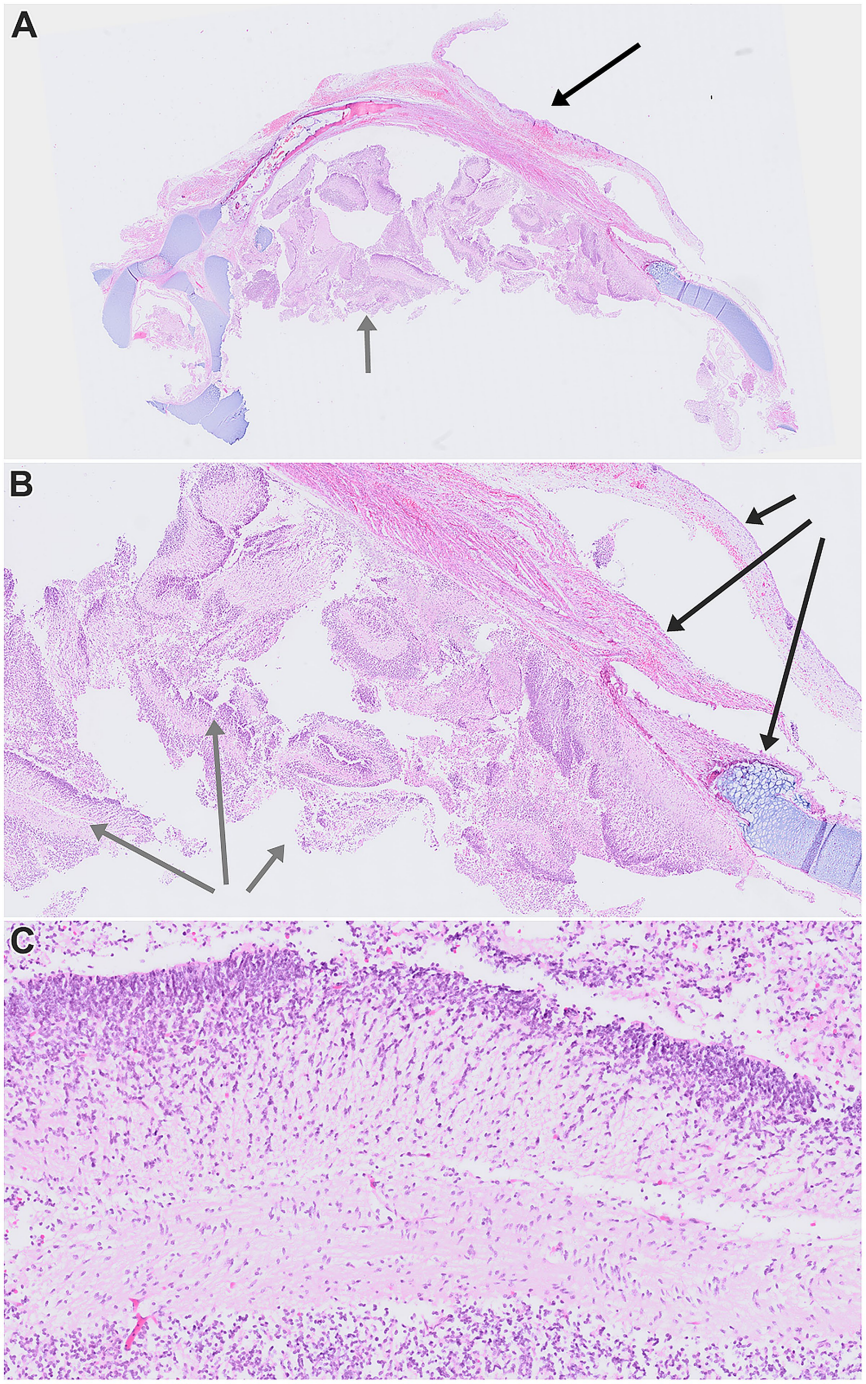
Scanned histopathological images of dissected chest wall tissues. **(A)** Overview at 0.46 × magnification, showing skin, soft tissues, immature cartilage structures, and heterotopic neuroglial elements. **(B)** The same area at 1.31 × magnification, highlighting the relationship between the thoracic wall and partially friable immature neuroglial tissue. **(C)** Detailed view of the heterotopic neuroglial tissue at 7.05 × magnification. In images **A** and **B**, black arrows indicate thoracic wall structures, while gray arrows mark heterotopic neuroglial tissue. Stained with hematoxylin-eosin.

Fetal autopsy was performed at the Institute of Clinical and Molecular Pathology and Medical Genetics. The fetus appeared appropriate for its gestational age. Measurements included a crown–heel length of 11 cm (normally 12.2–14.6 cm), crown–rump length of 8.5 cm (normally 8.9–10.7 cm), bilateral medial canthus distance of 0.5 cm, and lateral canthus distance of 2 cm from the midline. Head circumference was 7 cm (normally 8.4–10.8 cm), thoracic circumference was 8 cm (normally 8.6 cm), and abdominal circumference measured 5 cm (normally 6.9 cm). The medial edge of the foot measured 1 cm, while the lateral edge was 1.1 cm (both normally 1.4–1.6 cm). Both lower limbs exhibited signs consistent with bilateral clubfoot deformity. The fetal weight was recorded at 30 g (normally 34.6–63.6 g). Examination of the head, including the fontanelles, cranial bones, and dura mater, was unremarkable. A perpendicular section was made in the area of the posterior cerebral fossa, where the individual structures were severely autolysed. Due to the brain’s consistency, even after fixation, it was not feasible to unequivocally identify the presence of a congenital developmental abnormality. Upon opening the thoracic cavity, a gray mass measuring 1.5 × 1 cm was identified on the right side, arising from the chest wall and resembling neuroglial tissue (Figure 2). This mass compressed the lungs, leading to bilateral pulmonary hypoplasia. Cardiac examination revealed a ventricular septal defect alongside pulmonary artery hypoplasia. No other cardiac abnormalities were noted. The diaphragm and the abdominal organs appeared normal without any pathological findings. The placenta weighed 40 g (normally 41 g), with dimensions of 5 cm × 1 cm (normally 5.6 cm x 1 cm), and the centrally inserted umbilical cord measured 7.5 cm (normally 18.8 cm) in length, both of which were unremarkable. The histological examination of the placenta revealed no significant pathology. The chorionic villi were without signs of partial hydatidiform mole—no hydropic changes (without the formation of cisternae)—and trophoblast proliferation, pseudoinclusions, or mineralization was detected. The surface of the villi was more wrinkled in places, and finger-like processes were sporadically present. Immature intermediate villi predominated. Histopathological analysis of the thoracic mass confirmed the presence of immature neuroglial tissue, characterized by direct continuity of immature neuroepithelial structures with the thoracic cavity wall, including focal rosette-like formations (Figure 3). Additionally, examination of the occipital region revealed immature bone and soft tissues of the skull base and neck, with focal areas of immature meninges, brain tissue, and choroid plexus. These findings are suggestive of a cystic CNS malformation, possibly of the Dandy–Walker type. Subsequently, a genetic examination of the fetus was ordered and a chromosomal triploidy, (69, XXY), was found. No other genetic abnormalities were discovered.

The patient and her partner also underwent a genetic examination, with no abnormalities found, which would justify advising against further pregnancy. Their third attempt resulted in a missed abortion as well; however, on the fourth try, the pregnancy was uneventful and the patient gave birth to a healthy baby without any complications.

## Discussion

To our knowledge, this is the only reported case of subpleural neuroglial tissue, as opposed to neuroglia within the pulmonary tissue, and the only one described in a triploid fetus (1). Our case report could provide new insights into prenatal pathology and may serve as a foundation for further studies aimed at a better understanding of this rare pathology and its clinical implications. Given the limited number of cases of heterotopic neuroglial tissue in the thoracic cavity that have been described to date, we believe that our article will contribute to elucidating the pathogenesis of neuroglial heterotopies.

While heterotopic neuroglial tissue is exceedingly rare, instances of non-teratomatous extracranial heterotopias have been reported in paraneuraxial locations such as the nose, orbit, palatinal, and submandibular regions, often occurring in patients without a severe CNS defect. Neuroglial heterotopic lesions remote from the neuroaxis have been reported in the lung, where the vast majority of cases have been associated with failures of neural tube closure (5). Most of the reported cases describe either an anencephalic fetus or twins, where one sibling is anencephalic (Table 1), while the currently presented case is not associated with anencephaly. However, our case report appears to be the first to describe a fetus where the neuroglial heterotopia is associated with a possible Dandy–Walker malformation.

The Dandy–Walker malformation is one of the most frequent structural brain malformations, after other nosological units such as neural tube defects, Chiari malformations, hydrocephalus, or holoprosencephaly. The most common cerebellar malformation is the Dandy–Walker malformation, defined by hypoplasia and upward rotation of cerebellar vermis, a cystic enlargement of the fourth ventricle, and an enlarged posterior fossa. It is associated with an increased frequency of other CNS malformations, neural tube defects, heart defects, and cleft lip and/or palate (6).

Another unique association is that the presented fetus was completely triploid (69, XXY). Triploidy is a rare chromosomal abnormality characterized by an extra haploid set of chromosomes. It occurs in approximately 1% of conceptions, and many of the triploids are aborted spontaneously in the first trimester. Survival of the fetus beyond the first trimester is rare. Affected fetuses have been reported to have many malformations, such as holoprosencephaly with a median cleft lip, encephalocele, syndactyly, clubfeet, and heart defects. The most common malformation associated with it is syndactyly, but there are no obligate clinical features. As in our case, it is typically an incidental diagnosis found during a genetic examination offered to women with abnormal fetuses identified by a screening program (7, 8). Theoretically, the prevalence of neuroglial heterotopia associated with triploidy may be underestimated, as limited studies have been conducted, and the genotype is not routinely examined. Nonetheless, Alonso et al. reported a case of neuroglial heterotopia associated with multiple congenital anomalies, including microcephaly, café-au-lait spots, strabismus, thumb hypoplasia, incurving of the fifth digit, horseshoe kidney, and a duodenal membrane. The heterotopia manifested itself as four cystic nodules within the bronchial tree. Given that the infant was part of a twin pregnancy, the authors conducted DNA analysis on the heterotopic tissue to determine its origin. Their investigation revealed that the heterotopic tissue originated from a twin with congenital anomalies rather than the healthy twin (9).

Several theories have been proposed to explain the pathogenesis of neuroglial heterotopia in the lungs. These theories include vascular embolisation and implantation, following a head trauma, aberrant migration and differentiation of neural cells, teratoma, aspiration, and implantation of neuroglial tissue in fetuses with neural tube defects. Vascular embolism of brain tissue is the result of brain injury and may occur at any age. The neuroglial tissue can be found inside the blood vessels. However, death typically occurs shortly after the injury, thus leaving no time for successful implantation (5, 10). According to Gonzales-Cruzi et al., a teratomatous nature of the lesion can be excluded, as they are usually seen in older individuals, often contain more than one type of tissue, and are rarely multifocal (11).

Aspiration and implantation theory is therefore among the most favored ones, supported by cases of anencephalic twins. The theoretical reasoning is that due to the failure of skull development in anencephaly, the disorganized brain tissue is dislodged into the amniotic fluid, where it can be aspirated by the other twin (12). Although this theory is supported by the peribronchiolar localisation of lesions in patients with anencephalic twins or anencephalic patients, it may not fully explain cases like ours, where the heterotopic tissue grows subpleurally, outside the bronchial tree. Therefore, analogous cases might require a different theory to explain the localisation of neuroglial heterotopia. If that were the case, the fetus in this case report, who presented with a CNS malformation, would also have exhibited a peribronchial lesion. However, the localisation of neuroglial heterotopia in this instance suggests the need for an alternative explanation. In this case, the heterotopic tissue developed subpleurally, outside the lung tissue, exerting pressure on it.

It is also important to note the findings of a study by Peres et al., who aimed to ascertain the prevalence of brain tissue aspiration in children with neural tube defects. This study encompassed stillborn children and abortions and revealed GFAP-positive cells in the bronchioles in only 1 of the 22 cases examined. However, no heterotopy was found in the lung interstitium, indicating that while neuroglial cells may enter the lung, they do not necessarily implant and grow further. The authors concluded that while aspiration is uncommon, it does not inevitably lead to heterotopia (13). On the other hand, a study by Quemelo et al. aimed to assess the ability of glial cells to express molecules necessary for successful implantation. The study was performed on pregnant female Swiss mice. One fetus of each pregnant dam was removed by hysterotomy, its brain was disaggregated, and then implanted into the thorax of its siblings. They concluded that brain tissue heterotopia during fetal and postnatal period is able to complete integration and implantation (14). However, in the case report put forth by Alonso et al., with regard to the aforementioned theory, the authors took advantage of the different sexes of the twins and performed a genetic analysis to determine whether the heterotopia originated in the patient’s twin. Surprisingly, the lesion’s DNA showed that this was not the case (9).

The aberrant migration and differentiation theory, while speculative, stands as the sole explanation for the occurrence of heterotopia in children with no neural tube defects or traumas. On the other hand, owing to the rarity of the lesion, the only thing that can be stated with certainty is that the pathogenesis is unknown (5, 11). The theory of abnormal migration and differentiation of neural crest elements would explain both malformation of the brain and the neuroglial heterotopia. This theory can be supported to some extent by the association with the heart defect or triploidy described in our case. The aberrant migration could be a consequence of disrupted signaling pathways or altered gene expression patterns due to triploidy. The infant presented by Alonso et al., in addition to glial heterotopia, suffered from Fanconi anemia, a rare genetic disorder characterized by impaired DNA repair and genomic instability (9). Both Fanconi anemia and triploidy could impact DNA repair mechanisms, potentially leading to the misplacement of neuroglial tissue during embryonic development. Such genomic instability could, in turn, disturb the tightly regulated programs that guide neural crest migration. Genes such as SOX10, PAX3, and EDN1 are central to this process, directing neural crest cell differentiation, motility, and survival (15–17). The disruption of these pathways provides a plausible mechanistic link between chromosomal instability and aberrant migration. However, it must be emphasized that no specific genes have yet been identified as consistently overexpressed or causally linked to neuroglial heterotopy.

## Conclusion

In conclusion, our report unveils the first documented case of neuroglial heterotopia concomitant with both a probable Dandy–Walker malformation and triploidy. These unique associations lend credence to the plausibility of the aberrant migration and differentiation of neural crest elements theory as a potential pathogenesis. However, to confirm the theory, more reports, subsequent analyses, and studies are needed.

## Data Availability

The original contributions presented in the study are included in the article/Supplementary material, further inquiries can be directed to the corresponding author.
