# Supplementary figures and images for: When neuroglial tissue wanders: a unique case report of subpleural heterotopia in a triploid foetus and review of the literature
Source: Front Med (Lausanne). 2025 Sep 24;12:1598144. doi: 10.3389/fmed.2025.1598144 (PMC12506675; doi:10.3389/fmed.2025.1598144)

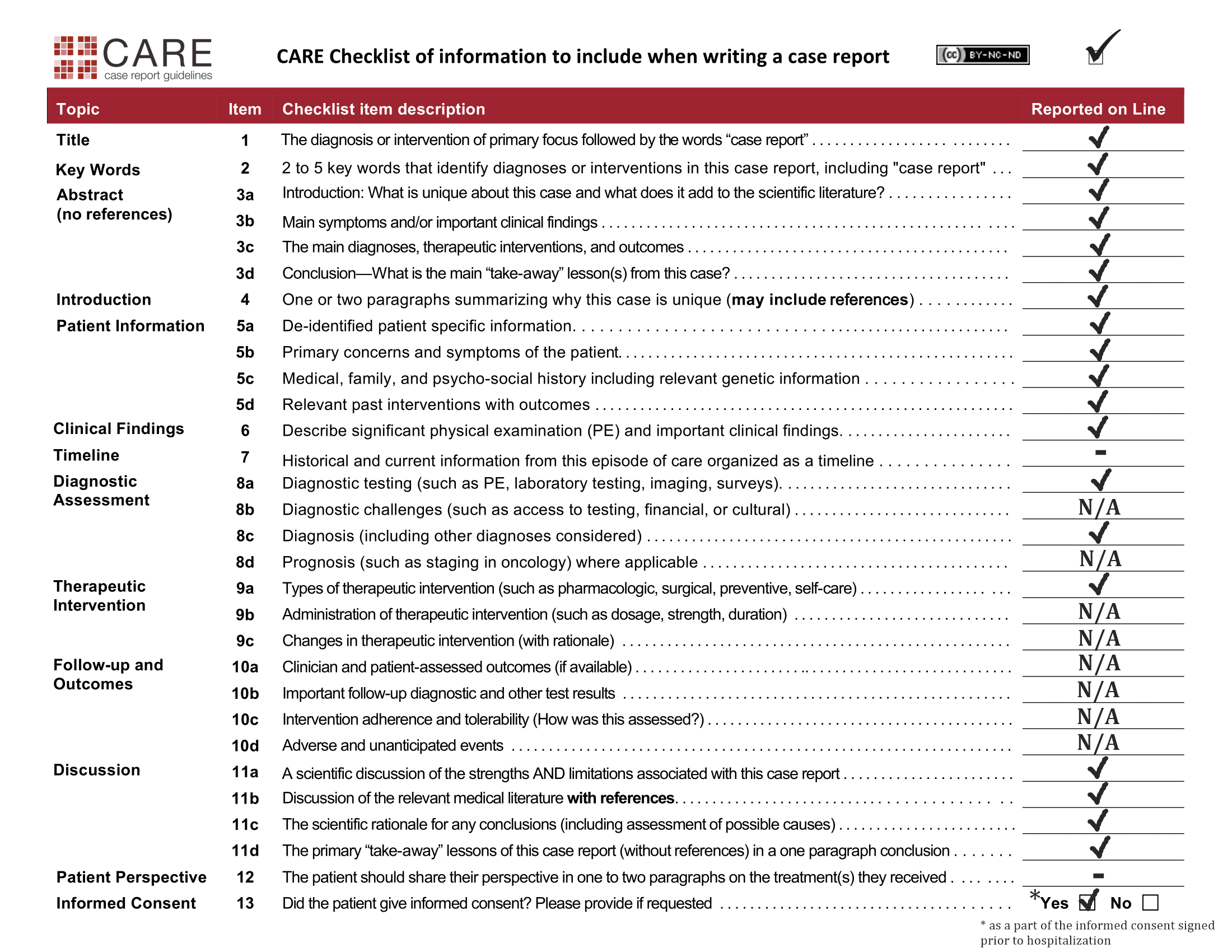

Supplement: Supplementary file 1 [file Image_1.PNG]
